# Role of KCa3.1 Channels in Modulating Ca^2+^ Oscillations during Glioblastoma Cell Migration and Invasion

**DOI:** 10.3390/ijms19102970

**Published:** 2018-09-29

**Authors:** Luigi Catacuzzeno, Fabio Franciolini

**Affiliations:** Department of Chemistry, Biology and Biotechnology, University of Perugia, 06134 Perugia, Italy

**Keywords:** KCa3.1 channels, glioblastoma, cell migration, calcium oscillations, mathematical model

## Abstract

Cell migration and invasion in glioblastoma (GBM), the most lethal form of primary brain tumors, are critically dependent on Ca^2+^ signaling. Increases of [Ca^2+^]_i_ in GBM cells often result from Ca^2+^ release from the endoplasmic reticulum (ER), promoted by a variety of agents present in the tumor microenvironment and able to activate the phospholipase C/inositol 1,4,5-trisphosphate PLC/IP_3_ pathway. The Ca^2+^ signaling is further strengthened by the Ca^2+^ influx from the extracellular space through Ca^2+^ release-activated Ca^2+^ (CRAC) currents sustained by Orai/STIM channels, meant to replenish the partially depleted ER. Notably, the elevated cytosolic [Ca^2+^]_i_ activates the intermediate conductance Ca^2+^-activated K (KCa3.1) channels highly expressed in the plasma membrane of GBM cells, and the resulting K^+^ efflux hyperpolarizes the cell membrane. This translates to an enhancement of Ca^2+^ entry through Orai/STIM channels as a result of the increased electromotive (driving) force on Ca^2+^ influx, ending with the establishment of a recurrent cycle reinforcing the Ca^2+^ signal. Ca^2+^ signaling in migrating GBM cells often emerges in the form of intracellular Ca^2+^ oscillations, instrumental to promote key processes in the migratory cycle. This has suggested that KCa3.1 channels may promote GBM cell migration by inducing or modulating the shape of Ca^2+^ oscillations. In accordance, we recently built a theoretical model of Ca^2+^ oscillations incorporating the KCa3.1 channel-dependent dynamics of the membrane potential, and found that the KCa3.1 channel activity could significantly affect the IP_3_ driven Ca^2+^ oscillations. Here we review our new theoretical model of Ca^2+^ oscillations in GBM, upgraded in the light of better knowledge of the KCa3.1 channel kinetics and Ca^2+^ sensitivity, the dynamics of the Orai/STIM channel modulation, the migration and invasion mechanisms of GBM cells, and their regulation by Ca^2+^ signals.

## 1. The Glioblastoma

The large majority (more than 90%) of cancer deaths are due not to the primary tumor per se, but to relapses arising from new foci established in distant organs via metastasis [1]. Glioblastoma (GBM), the most common and aggressive form of primary brain tumors, is no exception, though it does not metastasize in the classical way (that is, by colonizing other tissues via the bloodstream), but invades brain parenchyma by detaching from the original tumor mass and infiltrating into the healthy tissue by degrading the extracellular matrix or squeezing through the brain interstitial spaces. The urgency of tackling the migration and invasion issues of GBM tumors is clear.

The 2016 World Health Organization classification of the various types of brain tumors regards the presence of isocitrate dehydrogenase gene (*IDH1/2*) mutations as one of the most critical biomarkers. Accordingly, *IDH1/2* wildtype gliomas are categorized as glioblastoma (formerly primary glioblastoma) and *IDH1/2*-mutated gliomas (including formerly classified secondary glioblastoma) as astrocytic glioma and oligodendroglioma. GBMs are further subdivided into four groups (Proneural, Neural, Classical, and Mesenchymal), mainly based on the abnormally high levels of mutated genes (i.e., *EGFR* is highly upregulated in >98% of Classical GBM, whereas *TP53* (p53), which is most frequently mutated in Proneural GBM (50–60% of patients) is rarely mutated in Classical GBM). In spite of the intensive basic and clinical studies carried out over the past decades, and modern diagnostics and treatments, the average life expectancy for GBM patients is still only around 15 months. The major obstacle with GBM remains its high migratory and invasive potential into healthy brain parenchyma, which prevents complete surgical removal of tumor cells. Even with full clinical treatment (temozolomide-based chemotherapy and radiation therapy), tumors normally recur at some distance from the site of resection, establishing new tumor lesions that are by far the primary cause of mortality in GBM patients. Arguably, at the time of surgery, large numbers of cells have already detached from the original tumor mass and invaded normal brain tissue. Although GBM cell migration and invasion have been deeply investigated, many aspects of these processes are still poorly understood.

GBM cell migration is a highly regulated multistep process that initiates with GBM cells losing adhesion with surrounding elements, avoiding the cell death often associated with extracellular matrix (ECM) disconnection, and acquiring a highly migratory phenotype, which is a critical feature of the invasive process. The basic mechanisms underlying migration of GBM cells are common to most types of migratory cells. Migration is a property of many non-tumor cells, although it is often restricted to specific developmental stages or environmental conditions; the migration of tumor cells could be viewed as the result of mutation-induced dysregulation of specific biochemical pathways that in healthy tissue keep cell migration dormant.

## 2. Glioblastoma Cell Migration and Ca^2+^ Signaling

### 2.1. Cell Migration

The basic mechanisms of cell locomotion are now fairly well established. Locomotion can be described as the cyclical repeating of two main processes: (i) protrusion of the cell front due to local gain of cell volume mostly generated by active Na^+^/K^+^/2Cl^−^ cotransport accompanied by isoosmotically obliged water, and actin polymerization, with formation of pseudopods; (ii) retraction of the rear cell body in the direction of motion, due to forces produced by actomyosin contraction, accompanied by loss of cell volume generated by passive ion (mainly K^+^ and Cl^−^) fluxes and osmotic water [2,3]. These two processes involve the coordinated and localized formation of integrin-dependent cell adhesions at the leading edge, and their disassembly at the cell rear [4,5].

Protrusion of the cell front is sustained by localized polymerization of submembrane actin-based cytoskeleton that generates the pushing force and forms flat lamellipodia or needle-like filipodia. A large variety of signaling molecules have been shown to play a leading role in these processes, including the Rho GTPases family (that act as molecular switches to control downstream transduction pathways), and their effector proteins CDC42, RAC1, and RhoA. PI3 kinases have also been deeply implicated in controlling actin polymerization and lamellipodium extension. Activation of PI3 kinase by the pro-invasive signal molecules present in the tumor microenvironment functions as the trigger of the process, in that its activation initiates actin polymerization and generates membrane protrusion [5].

The retraction of the cell rear depends on the contractile forces generated by the activation of the myosin motors along crosslinked actin filaments. The myosin molecule is formed by two heavy chains that make up the two heads and the coiled-coil tail, and four—two essential and two regulatory—myosin light chains (MLCs). The myosin motor is primarily activated by a Ca^2+^-dependent cascade whereby a cytosolic Ca^2+^ increase activates a MLC kinase (MLCK) that leads to the regulatory MLC phosphorylation, which allows the myosin motor to crosslink with actin and produce tension to pull the rear end of the cell (however, some studies claim that the motor activity of myosin is not required for cell migration [6,7], playing instead a role in the establishment of cell polarity and in the coordination between different cell domains [7,8]). Clearly, to make the rear cell effectively move, the focal adhesions that anchor the actin cytoskeleton to the extracellular matrix (ECM) must be disassembled, normally through a proteolytic process that involves calpain, but also actin microtubules and focal adhesion kinase (FAK)-recruited dynamin that internalizes the integrins. Increasing actomyosin tension disrupts the possible residual resistances of focal adhesions at the cell rear [2]. Myosin can also be modulated by other pathways, including a Rho-dependent cascade (Rho → ROCK → MLC phosphorylation, or MLC phosphatase activation) that does not depend on cytosolic Ca^2+^.

This locomotion cycle can be initiated by a variety of external stimuli or directional cues which are sensed and decoded by specialized receptors on the plasma membrane and transferred to the cell interior via distinct signaling pathways to actuate the mechanical response. Most common migration stimuli rely on cells sensing (by specific G protein-coupled receptors) local gradients in the concentration of chemical factors (chemoattractants) [9] present in the tumor microenvironment (for instance chemokines IL-8 or CXCL12), or certain growth factors, including the platelet-derived growth factor (PDGF) that plays an important role in tumor metastasis [10]. Cancer cells can also be guided by gradients of bound ligands. Generally attached to the ECM, these ligands are recognized by specific integrins that form oriented focal adhesion to direct the formation of invadopodia, thus the direction of movement. Common is also the observation of cancer cells being guided by mechanical stimuli (mechanotaxis), namely the stiffness of the extracellular matrix they try to penetrate [11]. It is important to note that in vivo cells can be simultaneously subjected to multiple types of cues that need be evaluated as a whole in order to provide appropriate responses.

### 2.2. Cell Volume Changes Associated with Migration

It is important to recognize that these two steps—cell front protrusion and cell rear retraction—normally occur in succession, implying that cells are subjected to major changes in cell volume, especially the cell rear retraction. This requires compensatory ion fluxes (and osmotic water flow) across the plasma membrane in order to maintain proper cytosol osmolarity. Along the line proposed by [12] with regard to cell migration in general, during the protrusion of the cell front the maintenance of a stable osmolarity is sustained by cotransporters and ion exchangers, for instance Na^+^/K^+^/2Cl^−^ cotransport, followed by osmotically driven water. By contrast, the control of osmolarity during cell rear retraction critically depends on the activation of K channels and ensuing efflux of K^+^ ions (accompanied by Cl^−^ fluxes, to which the membrane has become highly permeant, and osmotic water). Following the opening of K channels the membrane potential hyperpolarizes to a value between the equilibrium potentials of Cl^−^ and K^+^ (E_Cl_ and E_K_), a condition that allows a significant efflux of KCl with consequent loss of water and cell volume decrease that facilitates cell body retraction. This view can explain the slowing of cell locomotion following the inhibition of K and Cl channels [13]. A number of papers indicate that the channel types primarily involved in osmolarity control are the intermediate conductance Ca^2+^-activated K channel (KCa3.1) and the Cl channel ClC3 [13,14].

### 2.3. Ca^2+^ Oscillations

Much work shows that cell migration is strictly regulated by Ca^2+^, which is no surprise given that many proteins involved in migration such as myosin, myosin light chain kinase (MLCK), Ca^2+^/calmodulin-dependent protein kinase II are Ca^2+^ sensitive [15]. In the resting cell Ca^2+^ concentration is kept very low (<100 nM) to prevent activation of Ca^2+^-sensitive proteins that act as Ca^2+^ sensor and initiation of unwanted biological processes. Ca^2+^ concentration can easily increase more than ten-fold (well over 1 µM) following several types of cell stimulation, and this would bring many of the major cytoplasmic Ca^2+^-sensitive proteins (calmodulin, PKA, Ca^2+^-activated channels, etc.) to activate. This opens the question of how Ca^2+^ signaling, under these conditions, can selectively activate a specific Ca^2+^ sensor protein and the associated biochemical cascade. Several strategies have evolved in this respect, but specificity is mostly attained by confining the signal at sub-cellular level, or by regulating kinetics and magnitude of the Ca^2+^ signal. Since in the cytoplasm Ca^2+^ diffusion is extremely low and buffering high, the opening of Ca^2+^ permeant channels results in a very localized Ca^2+^ increase (the Ca^2+^ microdomain), attaining spatial discrimination of the target proteins and linked downstream pathways. Another strategy relies on the temporal features of Ca^2+^ signals (rising rate, duration, repetition of spikes, etc.) that biological systems are capable to interpret.

In non-excitable cells, including GBM, typical Ca^2+^ signals induced experimentally by robust chemical (hormone) stimulation are slow, large and generally sustained. Hormones binding to their specific G-protein-coupled receptors (GPCRs) often results in the PLC-dependent synthesis of inositol 1,4,5-trisphosphate (IP_3_), and consequent Ca^2+^ release from the endoplasmic reticulum (ER) via IP_3_ receptors. Eventually, the sustained Ca^2+^ increase subsides as result of the activation of Ca^2+^ pumps placed on the sarco/endoplasmic reticulum (SERCA), or the plasma membrane (PMCA) that transfer Ca^2+^ ions from the cytosol to intracellular stores or the extracellular space.

A physiologically more meaningful type of Ca^2+^ signal, especially in relation to cell migration, where critical Ca^2+^-dependent processes are cyclical, comes in the form of Ca^2+^ oscillations. It has recently become clear that this type of oscillatory Ca^2+^ increases is what normally occurs when using physiological agonist stimulations. The mechanisms underlying Ca^2+^ oscillations are not fully understood, and often depend on the cell model and agonist used. In some systems Ca^2+^ oscillations are secondary to oscillations of the cytoplasmic IP_3_ level, which may be due to various types of feedback control that for brief intervals uncouple the GTP-coupled receptor from PLC. For instance, the reported negative feedback of the PLC products, IP_3_ and diacylglycerol (DAG), on PLC itself, or upstream on the GPCR, would result in IP_3_ oscillations [16], which would in turn generate cyclical release of Ca^2+^ from intracellular stores, and the Ca^2+^ oscillations [17].

**Figure 1:**
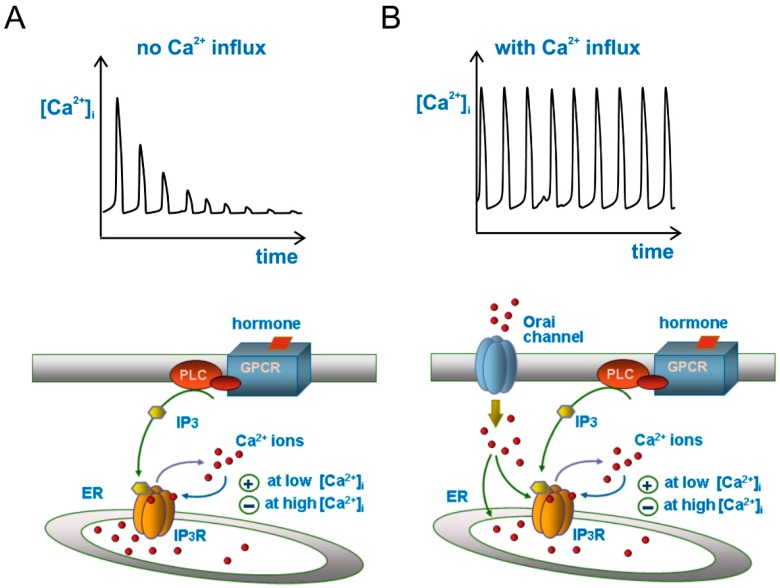
Ca^2+^ oscillations in response to inositol thriphosphate (IP_3_) increase, with and without Ca^2+^ influx from extracellular space. (**A**) Bottom, drawing illustrating the hormone-based production of IP_3_ that activates the IP_3_ receptor to release Ca^2+^ from endoplasmic reticulum (ER). The biphasic effects of cytosolic Ca^2+^ on IP_3_ receptor gating (the basic mechanism for Ca^2+^ oscillations), whereby Ca^2+^ modulates positively the receptor at low [Ca^2+^] but negatively at high [Ca^2+^], is also illustrated. Top, Ca^2+^ oscillations as produced from the schematics below. Note the decaying trend of Ca^2+^ spikes due to the absence of Ca^2+^ influx from extracellular space; (**B**) Here the drawing has been enriched with a Ca^2+^ influx apparatus from extracellular space through ER-depletion activated Orai channels on the plasma membrane (bottom), which generates sustained Ca^2+^ oscillations (top). For clarity, SERCA and PMCA Ca^2+^ pumps have not been sketched in the drawing, although their activity has always been taken into account. For the same reason, we omitted to draw STIM protein of the ER.

More commonly, however, Ca^2+^ oscillations can be observed in the presence of a constant level of IP_3_. The prevailing view is that, under these conditions, the repetitive Ca^2+^ oscillations secondary to moderate (physiological) hormone or agonist activation of membrane PLC and production of a constant amount of IP_3_ arises from the biphasic effects of cytosolic Ca^2+^ on the IP_3_ receptor gating, with the first phase being embodied by the establishment of the Ca^2+^-induced Ca^2+^ release (CICR) mechanism, whereby a moderate increase of [Ca^2+^] in the cytosol via IP_3_ receptor causes a positive feedback activation of the IP_3_ receptor that results in a higher Ca^2+^ released from internal stores ([18]; Figure 1A).

The second phase occurs when the cytosolic [Ca^2+^] reaches significantly higher levels, shifting the previous positive feedback on IP_3_ receptors into a delayed negative feedback that results in their closing and [Ca^2+^]_i_ returning to resting level by the action of both SERCA and PMCA Ca^2+^ pumps (Figure 1A). This view finds support from the observation that at constant levels of IP_3_, the activity of the IP_3_ receptor and the cytosolic Ca^2+^ concentration display a bell-shaped function, whereby low Ca^2+^ concentrations activate the receptor, whereas high Ca^2+^ concentrations inhibit it [19,20,21,22]. Ca^2+^ oscillations generated according to this scheme (i.e., Figure 1A) would however show a rapid decrease of spike amplitude with time, because part of the Ca^2+^ released from the ER during each spike will be pumped out of the cell by PMCA, and be no longer available for refilling the ER and contributing to the next Ca^2+^ spike. This Ca^2+^ oscillations time course is observed experimentally upon removal of external Ca^2+^, or blockade of Ca^2+^ influx from the extracellular space. To have sustained (or slow decaying) Ca^2+^ oscillations, external Ca^2+^ pumped out of the cell by PMCAs must be allowed to re-enter the cell. This is most commonly accomplished through the mechanism of store-operated Ca^2+^ entry (SOCE).

### 2.4. Store-Operated Ca^2+^ Entry (SOCE)

When Ca^2+^ is released from the ER, i.e., following hormone stimulation, the Ca^2+^ concentration within the organelle decreases. It is this decrease to trigger the process (known as SOCE) whereby Ca^2+^ channels in the plasma membrane are activated, allowing entry of Ca^2+^ into the cell. Although the process of SOCE was described some 30 years ago, its mechanisms and molecular counterparts—the STIM and Orai proteins—have been identified much more recently [23,24,25]. The stromal interaction molecules (STIMs) are a family of one-passage integral proteins of the ER that act as sensors of the Ca^2+^ concentration within the ER. Orai proteins form instead a group of highly selective Ca^2+^ channels placed in the plasma membrane. Orai channels are neither activated by voltage, nor by agonists (in the classical sense), and are normally silent when the cell is at rest and the ER filled with Ca^2+^ ions. Orai Ca^2+^ channels are opened by the interaction with activated STIM proteins, which occurs following depletion of Ca^2+^ in the ER, and their opening results in Ca^2+^ ions flowing inside the cell (the mechanism has been recently reviewed in depth on neurons by [26]). This Ca^2+^ entry into the cytoplasm enables the refilling of Ca^2+^ depleted ER through the SERCA pumps, and make the Ca^2+^ oscillations amplitude to be kept stable with time (Figure 1B). In the context of our review topic, this feedback mechanism of store-operated Ca^2+^ entry gains further relevance for two more reasons: it contributes to further modulating the IP_3_ receptor (this modulation is also dependent on the cell type—for instance, in U87-MG human GBM cells Ca^2+^ oscillations are affected only marginally by removal of Ca^2+^ influx [27], whereas in rat C6 GBM cells this maneuver fully suppresses Ca^2+^ oscillations [28]), and it represents the target on which the KCa3.1 channel exerts its modulation of Ca^2+^ oscillations, as we will see later.

It needs to be recalled that the store-operated Ca^2+^ entry is not the only suggested mechanism—although it is by far the most widely accepted—that links hormone stimulation to increased plasma membrane Ca^2+^ entry. For instance, heteromers of Orai1 and Orai3 can be activated by arachidonic acid [26], and the Ca^2+^-permeant TRPC6 by receptor-induced PLC activation via direct action of DAG. Notably, neither pathway involves IP_3_ receptor or Ca^2+^ depletion from the ER.

### 2.5. Ca^2+^ Oscillations and Cell Migration

Numerous studies have shown that migrating GBM cells display evident Ca^2+^ oscillations having a period of several tens of seconds, whose presence appears to be essential to the process of migration. For example, in U87-MG cells Ca^2+^ oscillations may be induced by the pro-migratory fetal calf serum (FCS), and trigger focal adhesion disassembly during cell rear retraction [29,30]. Similarly, in D54-MG cells prolonged exposure to bradykinin induces Ca^2+^ oscillations, which significantly enhance cell motility [31]. Mechanistically, the Ca^2+^ signal may translate into actomyosin contraction and rear cell retraction by first binding to calmodulin (CaM). The activated complex, Ca^2+^/CaM, then activates MLCK, the kinase that phosphorylates the regulatory myosin light chains (MLC), thereby triggering cross-bridge movements and actomyosin contraction. It has been shown that MLCK can be modulated by several other protein kinases including PKA, PKC, and RhoK, indicating that this critical enzyme for cell migration is under the influence and control of many extracellular signals and cytoplasmic pathways. Although the consensus on this mechanism and the role of MLCK is widespread, a little caution is needed as most studies used a pharmacological approach, but really specific MLCK inhibitors were not available [32]. A recent report showed, for instance, that knockout cells for MLCK maintained unaltered (or increased) their migratory ability [33]. This shows that if the critical role of Ca^2+^ in cell motility is undisputed, uncertainties may remain as to how Ca^2+^ is linked to the cell migratory machinery. The role of the Ca^2+^-activated K channels in cell volume changes and the dynamics of Ca^2+^ signals during migration may be a valid alternative.

## 3. The Intermediate Conductance Ca^2+^-Activated K Channel

The intermediate conductance Ca^2+^-activated K channel, KCa3.1, belongs to the Ca^2+^-activated K channel (KCa) family, which comprises the large- (KCa1.1), intermediate (KCa3.1), and small conductance (KCa2.1-3) K channels, as originally classified according to their single-channel conductance. Genetic relationship and Ca^2+^ activation mechanisms have later shown that these channels form two well-defined and distantly related groups. One, including only the large-conductance KCa1.1 channel, is gated by the cooperative action of membrane depolarization and [Ca^2+^]_i_, while the other group includes both the intermediate conductance KCa3.1 and small-conductance KCa2.1-3 channels, gated solely by cytosolic Ca^2+^ increase.

### 3.1. Biophysics, Pharmacology, and Gating of the KCa3.1 Channel

The biophysical properties of the KCa3.1 channel have been investigated using various experimental approaches and cell models. Work on cultured human glioblastoma cells shows high K^+^ selectivity, moderate inward rectification, and single-channel conductance of 20–60 pS in symmetrical 150 mM K^+^. The KCa3.1 channel is voltage-insensitive, but highly sensitive to [Ca^2+^]_i_ showing an EC_50_ of <200 nM. Ca^2+^ ions activate the KCa3.1 channel via the Ca^2+^-binding protein CaM, which is constitutively bound to the membrane-proximal region of the intracellular C terminus of the channel. Binding of Ca^2+^ to CaM results in conformational changes of the channel, and its opening. In addition to serving as a Ca^2+^ sensor for channel opening, CaM also regulates the assembly and trafficking of the channel protein to the plasma membrane [34,35].

The KCa3.1 channel can be blocked by peptidic toxins isolated from various scorpions, such as charybdotoxin (ChTx) and maurotoxin (MTx), which display high affinity (IC_50_ in the nM range). Small synthetic molecules have also been developed, many derived from the clotrimazole template, the classical KCa3.1 channel blocker. The most widely used TRAM-34, developed by Wulff’s group, inhibits KCa3.1 channels with an IC_50_ < 20 nM and displays high selectivity over the other KCa channels. As for KCa3.1 channel activators, we recall the benzimidazolone 1-EBIO that activates the channels with an EC_50_ < 30 μM and DC-EBIO that exhibits 10-fold higher potency. Two structurally similar and still more potent molecules are the oxime NS309 and the benzothiazole SKA-31, both showing an EC_50_ for KCa3.1 channels < 20 nM. We finally mention riluzole, another potent activator of KCa3.1 channels, which is the only FDA-approved drug for treatment of amyotrophic lateral sclerosis (ALS).

### 3.2. KCa3.1 Channel Expression and Impact on GBM Migration and Invasion

Initial biochemical and electrophysiological studies showed that KCa3.1 channels were diffusely expressed in virtually all cell types investigated. Notably, the KCa3.1 channel was not found in the brain (later the KCa3.1 channel was described in microglia [7], several types of brain neurons [8,36], and the nodes of Ranvier of cerebellar Purkinje neurons [37]), although it was highly expressed in established cell lines from brain tumors, as well as in brain tumors in situ. Moreover, KCa3.1 channel expression was reported in many other tumor types, and implicated in malignancy (increased cell growth, migration, invasion, apoptosis evasion). The KCa3.1 mRNA and protein expression were also found to be significantly enhanced in cancer stem cells derived from both the established cell line U87-MG and primary cell line FCN9 [38]. The REMBRANDT database shows that the gene KCNN4, which encodes the KCa3.1 channel, is overexpressed in more than 30% of gliomas, and its expression is associated with a poor prognosis.

To test the role of the KCa3.1 channel in GBM cells’ infiltration into brain parenchyma, human GL-15 GBM cells were xenografted into the brains of SCID mice and later treated with the specific KCa3.1 blocker TRAM-34. Immunofluorescence analyses of cerebral slices after a five-week treatment revealed a significant reduction of tumor infiltration, compared with TRAM-34 untreated mice [39]. Reduction of tumor infiltration was also observed in the brain of mice transplanted with KCa3.1-silenced GL-15 cells, indicating a direct role of KCa3.1 channels in GBM tumor infiltration [40]. Similarly, KCa3.1 channel block with TRAM-34 or silencing by short hairpin RNA (shRNA) completely abolished CXCL12-induced GL-15 cell migration [40]. A strong correlation of KCa3.1 channel expression with migration was reported by Calogero’s group in GBM-derived cancer stem cells (CSC) [38]. Blockage of the KCa3.1 channel with TRAM-34 was found to have a much greater impact on the motility of CSCs (75% reduction) that express a high level of KCa3.1 channel than on the FCN9 parental population (32% reduction), where the KCa3.1 channel is expressed at much lower levels. Similar results were also observed with the CSCs derived from U87-MG [38] and with mesenchymal glioblastoma stem cells [41]. On the same line stand the results later obtained by Sontheimer’s group showing that pharmacological inhibition of the KCa3.1 channel (with TRAM-34) in U251 glioma cells, or silencing it with inducible siRNA, resulted in a significant reduction of tumor cells’ migration in vitro and invasion into surrounding brain parenchyma of SCID mice [42]. On a retrospective study of a patient genomic database, they further showed that KCa3.1 channel expression was inversely correlated with patient survival.

### 3.3. Basic Functions of KCa3.1 Channel: Regulation of Cell Ca^2+^ Signaling

KCa3.1 channels control a number of basic cellular processes involved in the modulation of several higher-order biological functions critical to brain tumors’ malignancy, including migration and invasion. The most relevant and common basic cellular processes controlled by KCa3.1 channels are the modulation of Ca^2+^ signaling and the control of cell volume. Here we will concentrate on the KCa3.1 channel modulation of Ca^2+^ signaling, the focus of this review.

In virtually all cells a robust stimulation of PLC-coupled membrane receptors triggers an initial IP_3_-mediated release of Ca^2+^ from intracellular stores, followed by Ca^2+^ influx through store-operated Orai Ca^2+^ channels, which are activated in response to Ca^2+^ depletion of the ER. One consequence of this Ca^2+^ influx, besides the activation of Ca^2+^-dependent target proteins (ion channels included), is membrane depolarization, which, if left unchecked, would more and more strongly inhibit further Ca^2+^ influx due to the reduced electrochemical driving force for Ca^2+^ ions. It has been shown that the efflux of K^+^ ions following KCa3.1 channels’ activation by incoming Ca^2+^ hyperpolarizes the membrane towards E_K_, increasing the electromotive force on incoming Ca^2+^ ions [43,44]. This represents an indirect modulatory mechanism of Ca^2+^ entry and, as a result, of cell Ca^2+^ signaling. The Ca^2+^-dependent and voltage-independent gating of KCa3.1 channels appears to be particularly well suited for this function, since the coupling of Ca^2+^ influx and activation of K^+^ channels will create a positive feedback loop whereby more Ca^2+^ influx will increase the K^+^ efflux, hyperpolarize the plasma membrane, and further stimulate Ca^2+^ entry, thus amplifying the signal transduction.

This paradigm was first demonstrated in human macrophages, where KCa3.1 channels have been shown to hyperpolarize the membrane and increase the driving force for Ca^2+^ ions during store-operated Ca^2+^ entry [43], and in T cells, where KCa3.1 channels are rapidly upregulated following cell activation, and supposedly used to maximize Ca^2+^ influx during the reactivation of memory T cells [45,46]. Activated T cells isolated from KCa3.1^−/−^ mice show a defective Ca^2+^ response to T cell receptor activation [47]. Also, mast cells appear to use KCa3.1 channels to hyperpolarize the membrane and increase the Ca^2+^ influx following antigen-mediated stimulation. In this case KCa3.1 channels have been found to physically interact with the Orai1 subunit, suggesting that they may be activated by the Ca^2+^ microdomain that forms close to the store-operated Ca^2+^ channel [28,48]. A similar conclusion has been reached for rat microglial cells, where the Orai1-KCa3.1 functional coupling could be interrupted by BAPTA but not by EGTA [49]. Since the two Ca^2+^ chelators have similar Ca^2+^ affinity but different Ca^2+^ binding rates (10 times higher for BAPTA), only a physical coupling between Orai1 and KCa3.1, with a separating distance of few nanometers, would explain these results.

Evidence of cross-talk between the KCa3.1 channel and store depletion-activated Ca^2+^ influx has also been reported for GBM cells. An increase of [Ca^2+^]_i_, consisting of a fast peak due to IP_3_-dependent Ca^2+^ release from intracellular stores followed by a sustained phase of Ca^2+^ influx across the plasma membrane, supposedly activated by intracellular stores’ depletion, was found upon prolonged application of histamine on GL-15 GBM cells [44]. The enhancing role of the KCa3.1 channel in sustaining the protracted influx of external Ca^2+^ was shown by the marked reduction of the sustained histamine-induced [Ca^2+^]_i_ increase following application of TRAM-34. This observation could be significant with regard to the KCa3.1 channels’ contribution to GBM cell migration exerted through modulation of Ca^2+^ signals.

In another GBM cell line—U87-MG—we also observed that the promigratory FCS is able to promote Ca^2+^ oscillations, and these oscillations cyclically activate the KCa3.1 channels during cell migration [13]. Using a modeling approach, we also found that a channel activity with the properties of KCa3.1 channels could modulate these Ca^2+^ oscillations (it increased the amplitude, duration, and frequency of each Ca^2+^ spike [50]). The results we obtained, which are illustrated in the following section, were unexpectedly interesting, and also able to explain old experiments showing that the KCa3.1 channel inhibition by ChTx abolishes the bradykinin-induced Ca^2+^ oscillations in C6 glioma cells [51].

### 3.4. Modulation of Ca^2+^ Oscillations by KCa3.1 Channel Activity

We saw earlier that the mechanism underlying Ca^2+^ oscillations is essentially based on the biphasic effects of Ca^2+^ on IP_3_ receptor gating—activatory at low and inhibitory at high concentrations. We also discussed the modulation that the KCa3.1 channel could exert on Ca^2+^ oscillations. This modulation relies on the high Ca^2+^ sensitivity and voltage independence of the KCa3.1 channel, and on the output of its activity, that is, the hyperpolarization of the cell membrane potential (*V*_m_) as result of the K^+^ efflux. As KCa3.1 channels are activated by Ca^2+^ concentrations well within the range observed during hormone-induced Ca^2+^ oscillations (150–600 nM; cf. above), they are expected to cyclically open (during the Ca^2+^ spikes) and cause cyclic membrane hyperpolarizations in phase with the Ca^2+^ oscillations.

We have observed experimentally such KCa3.1 channel oscillatory activity and associated membrane potential oscillations in U87-MG cells in response to FCS [13], an agent known to induce Ca^2+^ oscillations in these cells [29,30]. Notably, because of their voltage dependence and much lower Ca^2+^ sensitivity, the KCa1.1 channels, also highly expressed in GBM cells, could not be activated under these conditions, even at the peak Ca^2+^ concentration of the oscillations ([13], but see [52] for a case in which the KCa1.1 channel activity may control Ca^2+^ influx). In conclusion, since *V*_m_ controls the driving force for external Ca^2+^ entry, KCa3.1-dependent *V*_m_ oscillations are expected to cause oscillations in the amplitude of Ca^2+^ influx through the hormone-activated plasma membrane Ca^2+^ channels, which will in turn feedback onto, and modulate the Ca^2+^ oscillations themselves.

**Figure 2:**
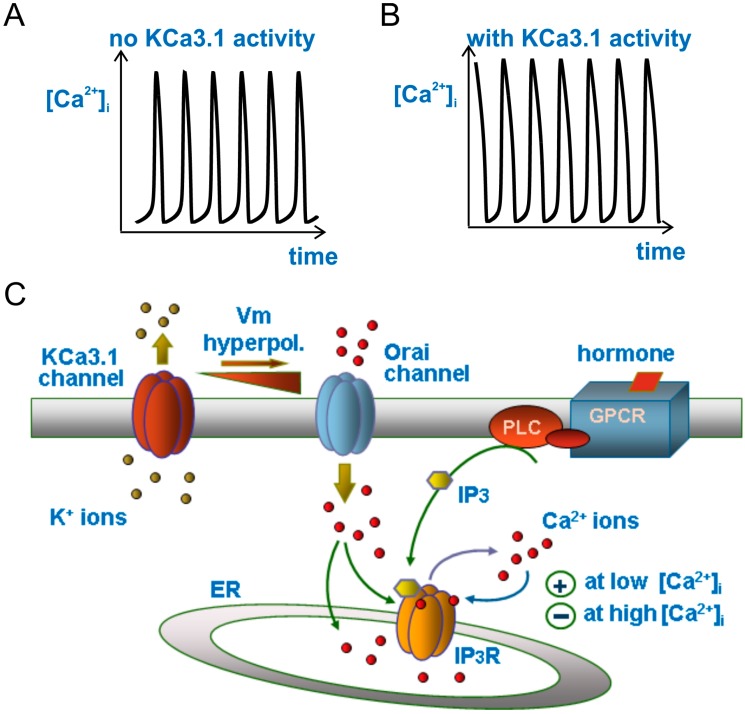
Modulation of Ca^2+^ oscillations by the KCa3.1 channel. Drawing (**C**) illustrating the main ion fluxes generating or modulating the Ca^2+^ oscillations in our model when the KCa3.1 channel is cut off (**A**) or introduced into the system (**B**). Please notice the much longer duration (width) and slightly higher amplitude of the Ca^2+^ oscillations in the presence of KCa3.1 channels.

We recently implemented basic theoretical models of Ca^2+^ oscillations with the oscillating membrane *V*_m_, as produced by the cyclic activation of KCa3.1 channels. The resulting model was used to predict how the hormone-induced Ca^2+^ oscillations would be influenced by the KCa3.1 channels-induced *V*_m_ oscillations in phase with Ca^2+^ oscillations. We found that the cyclic activation of KCa3.1 channels by Ca^2+^ oscillations induces *V*_m_ fluctuations that in turn determine oscillations in the Ca^2+^ influx from the extracellular medium, as a result of the oscillating *V*_m_-dependent changes in the driving force for Ca^2+^ ions influx. Since Ca^2+^ influx peaks are in phase with the Ca^2+^ oscillations, the Ca^2+^ spikes will also be increased by the in-phase Ca^2+^ influx. Our model calculations in fact show that KCa3.1-induced *V*_m_ oscillations strengthen the hormone-induced [Ca^2+^]_i_ signals by increasing the amplitude, duration, and oscillatory frequency of Ca^2+^ spikes (Figure 2; see also [50]).

### 3.5. KCa3.1 Channels Switch Ca^2+^ Oscillations On and Off

**Figure 3:**
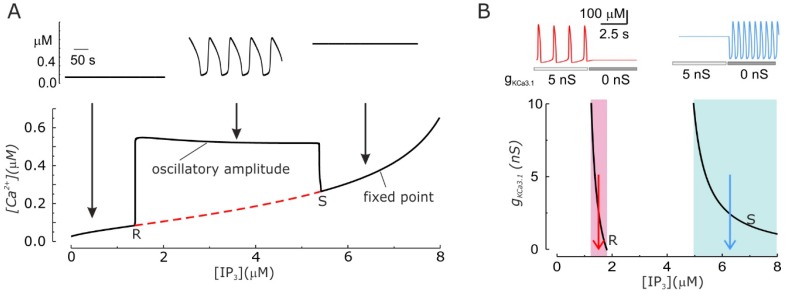
Ca^2+^ oscillation development depends on the IP_3_ concentration and the activity of KCa3.1 channels. (**A**) Bottom. Bifurcation diagram showing that IP_3_ concentration determines the establishment of the Ca^2+^ oscillations. The red dashed line represents the unstable equilibrium [Ca^2+^]_i_. The computation was performed with g_KCa3.1_ = 5 nS. R and S indicate the Hopf bifurcations where Ca^2+^ oscillations appear and disappear, respectively, upon increasing the IP_3_ levels. Top. The traces show the temporal changes of the [Ca^2+^]_i_ for three different concentrations of IP_3_ (indicated by arrows in the bottom part). (**B**) Bottom. Plot of the two Hopf bifurcations (R and S) as a function of the IP_3_ concentration and g_KCa3.1_. Three different ranges of IP_3_ concentrations, where the KCa3.1-induced *V*_m_ oscillations appear to have different effects in the modulation of Ca^2+^ oscillations are evidenced (shaded areas; see text). More specifically in the pink region the presence of KCa3.1 channels is necessary for the existence of Ca^2+^ oscillations, in the light blue region KCa3.1 channels prevents Ca^2+^ oscillations, and finally in the white region in between KCa3.1 channels modulate the shape and duration of oscillation. Top. Simulated Ca^2+^ oscillations obtained with two IP_3_ concentrations (1.5 left and 6.2 μM right, as indicated by the red and blue arrows below) within the regions where removing KCa3.1 channels make Ca^2+^ oscillations disappear or appear. (Modified from [48].)

Arguably, the KCa3.1 channels can do more than just modulate the amplitude and frequency of Ca^2+^ oscillations: they can trigger or suppress them. This has been an unexpected prediction of our model that we later found to have been experimentally observed. As experimentally found, our model predicts that Ca^2+^ oscillations can be generated only within a specific range of IP_3_ concentrations, while a stable, non-oscillating Ca^2+^ level is present for both lower and higher IP_3_ concentrations (Figure 3A). Surprisingly, it was found that when the KCa3.1 conductance is changed in the system, the resulting alteration of the KCa3.1 channels-induced *V*_m_ oscillations significantly change the IP_3_ concentrations needed to produce Ca^2+^ oscillations. In particular, increasing the activity of KCa3.1 channels shifts leftward the IP_3_ range within which Ca^2+^ oscillations are produced. This implies that there are ranges of IP_3_ levels where the presence or absence of Ca^2+^ oscillations are only dictated by the activity (conductance) of KCa3.1 channels (Figure 3B bottom, shaded areas).

These observations could explain a number of experimental results where KCa3.1 channel modulation was shown to have an effect on Ca^2+^ oscillations. In both activated T lymphocytes and C6 glioma cells, inhibition of *V*_m_ oscillations by blocking KCa3.1 channels is able to suppress Ca^2+^ oscillations [51,53]. Moreover *ras*-induced transformation of fibroblasts, which has been reported to upregulate KCa3.1 channels, has also been shown to trigger Ca^2+^ oscillations [54,55]. Finally, KCa3.1 inhibition leads to induction of Ca^2+^ oscillations in about half of the tested glioblastoma cells [52].

### 3.6. Glioblastoma KCa3.1 Channels Are Activated by Serum-Induced Ca^2+^ Oscillations and Participate to Cell Migration

Glioblastoma cells in vivo are exposed to a variety of tumor microenvironment components and soluble factors that can markedly affect their migratory ability. Among them there are unknown serum components that infiltrate into the tumor area of high-grade gliomas as result of the blood brain barrier breakdown [6,56]. As already shown, FCS enhances migration of U87-MG glioblastoma cells, and does so by inducing Ca^2+^ oscillations that are critically involved in the detachment of focal adhesions (by activating the focal adhesion kinase), and subsequent retraction of the cell rear [29,30]. On the same cell model we further reported that FCS, besides Ca^2+^ oscillations, induces an oscillatory activity of KCa3.1 channels, and this KCa3.1 channel activity is a necessary step to promote U87-MG cell migration by FCS [13]. In the same study, we also found a stable activation of Cl^−^ currents upon FCS stimulation. Altogether these observations sketch a coherent picture of the cell migratory process, in that, the retraction of the cell rear after the detachment of focal adhesions involves a major cell shape rearrangement and volume reduction. In these instances, the (oscillatory) KCa3.1 channel activity and the consequent cyclical K^+^ efflux, in combination with Cl^−^ efflux (and essential osmotic water), form part of the whole machinery for achieving the cyclical volume reduction needed.

**Figure 4:**
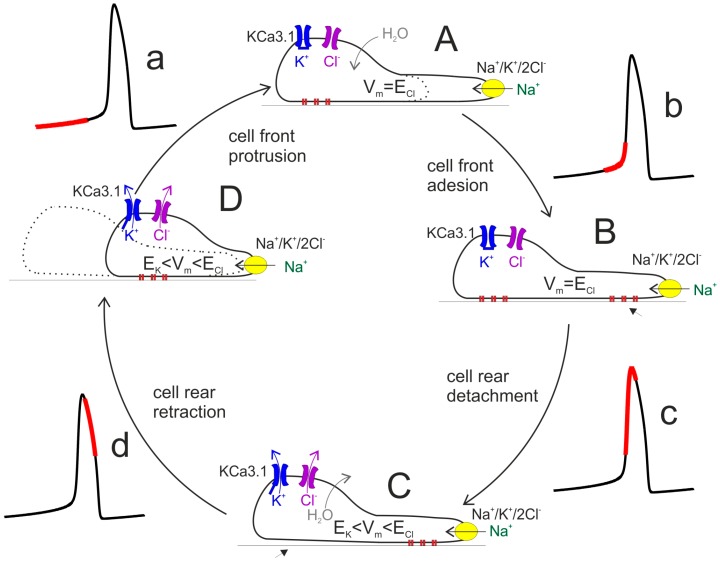
Schematic diagram of cell migratory cycle. The classic view of the cell migration process can be split down into four main cyclical steps. The cycle begins with the cell front protrusion, due to the activity of Na^+^/K^+^/2Cl^−^ cotransport (yellow) (**A**) and establishment of adhesion structures (**B**). The elongated cell then removes/weakens the rear adhesions (**C**) so that ensuing contraction can pull the rear cell portion forward (**D**). The concomitant values of [Ca^2+^]_i_ is indicated by the red portion on the associated Ca^2+^ oscillation. *V*_m_, ion and water fluxes are also illustrated (see text for details).

Cell locomotion is thought to be promoted by the cycling replication of four main steps: (i) protrusion of the cell front, associated to asymmetric polymerization of the actin-based cytoskeleton; (ii) adhesion of the protruded cell front to the substratum, mediated by the binding of focal adhesions to the extracellular polymers in contact with the cell; (iii) unbinding of the focal adhesions at the cell rear; (iv) retraction of the rear cell body in the direction of motion (cf. Figure 4). Two of these steps, namely cell front protrusion and cell rear retraction, involve major changes in cell volume and thus require compensatory ion fluxes (and osmotic water flow) across the membrane. Along the lines proposed by [12], during the protrusion of the cell front the maintenance of a stable osmolarity is sustained by cotransporters and ion exchangers—for instance, Na^+^/K^+^/2Cl^−^ cotransport—followed by osmotically driven water. During this phase [Ca^2+^]_i_ is relatively low (red segment in the Ca^2+^ oscillation, Figure 4a), a condition in which KCa3.1 channels are closed and the resting membrane potential is near E_Cl_. This condition prevents or strongly limits the loss of KCl via K and Cl channels that would nullify the osmotic work of the Na^+^/K^+^/2Cl^−^ cotransport.

By contrast, the control of osmolarity during cell rear retraction critically depends on the activation of KCa3.1 channels and ensuing efflux of K^+^ ions (accompanied by Cl^−^ fluxes, to which membrane has become highly permeant, and osmotic water). Following the increase in [Ca^2+^]_i_ towards the peak of the Ca^2+^ oscillation (Figure 4c,d), KCa3.1 channels open and the membrane potential hyperpolarizes to a value between E_Cl_ and E_K_, a condition that would allow a significant efflux of KCl with consequent loss of water and cell volume decrease (Figure 4C,D). This would facilitate the process of cell body retraction. This view can explain the slowing of cell locomotion following the inhibition of K and Cl channels [13].

## 4. Conclusions

Substantial evidence suggests that the oscillatory activity of the KCa3.1 channel during Ca^2+^ oscillations may have both direct and indirect modulatory effects on GBM cell migration. As has long been recognized, the K^+^ efflux through KCa3.1 channels directly participates in the cell volume changes during cell rear retraction. The same oscillatory K^+^ efflux, however, will also determine the membrane potential oscillations that alter the driving force for Ca^2+^ influx and indirectly modulate the properties and even the presence of Ca^2+^ oscillations, and thus the timing of the migratory machinery. Both modulatory roles make KCa3.1 channels pivotal in GBM cell migration, and thus potential pharmacological targets for this deadly tumor. Although this review concentrates on the role of KCa3.1 and STIM/Orai channels in modulating Ca^2+^ oscillations and cell migration, it needs to be said that other channels including TRPM8 and KCa1.1, also abundantly expressed in glioblastoma cells, may also be important in these processes. Accordingly, TRPM8 [41] and KCa1.1 channel signaling [57] have been demonstrated to be required for basal and radiation-induced migration, respectively, and certainly contribute to Ca^2+^ oscillations.
